# Poorly differentiated sarcoma of the maxillary sinus: a histopathology dilemma of a rare tumor

**DOI:** 10.1093/jscr/rjac504

**Published:** 2022-11-14

**Authors:** Tena Šimunjak, Boris Šimunjak, Martin Jurlina, Matea Zrno

**Affiliations:** Department of Otorhinolaryngology, Head and Neck Surgery, University Hospital Sveti Duh, Zagreb, Croatia; Department of Otorhinolaryngology, Head and Neck Surgery, University Hospital Sveti Duh, Zagreb, Croatia; Faculty of Dental Medicine and Health Osijek, Josip Juraj Strossmayer University of Osijek, Osijek, Croatia; Department of Maxillofacial Surgery, University Hospital Dubrava, Zagreb, Croatia; University of Zagreb, School of Medicine, Zagreb, Croatia; Department of Otorhinolaryngology, Head and Neck Surgery, University Hospital Sveti Duh, Zagreb, Croatia

## Abstract

Sarcomas are a rare heterogeneous group of neoplasms of mesenchymal origin. In the redistribution of all head and neck malignancies, sarcomas are represented by only 1%. Herein, we report a case of a 66-year-old patient with right maxillary sinus sarcoma that spread through the ostiomeatal complex, infiltrated the septum, all ethmoid cells, frontal sinus, involved the entire right nasal cavity and penetrated to the nasopharynx. Patient was treated with neoadjuvant chemotherapy, surgery and adjuvant radiotherapy. The histopathology indicated poorly differentiated sarcoma with elements of Ewing’s sarcoma, but also with elements consistent with osteosarcoma. Molecular pathological analysis excluded Ewing's sarcoma. Samples were also sent for review to the other Pathology Clinics. They suggested poorly differentiated high-grade pleomorphic sarcoma with elements of osteosarcoma. The accurate diagnosis of the head and neck sarcoma type can be a histopathology dilemma posing a great challenge in the choice of therapeutic approach, and thus the treatment outcome.

## INTRODUCTION

Sarcomas are rare heterogeneous group of neoplasms of mesenchymal origin. The majority (80%) of head and neck sarcomas are histogenetically soft tissue origin, and only 20% are of bone origin. In the redistribution of all head and neck malignancies, all sarcomas are represented by only 1–2% [1]. The etiology of sarcoma has not been fully elucidated, but several environmental and genetic factors have been associated with the development of sarcomas. Non-randomized chromosomal translocations that lead to the formation of oncogenic proteins, telomere and tumor suppressor genes dysfunction underlie in the development of sarcoma [2]. Paranasal sinuses sarcomas are extremely rare, including data on the involvement of a single paranasal sinus, as the literature is limited to sporadic cases and smaller series of individual subtypes [3].

## CASE REPORT

**Figure 1:**
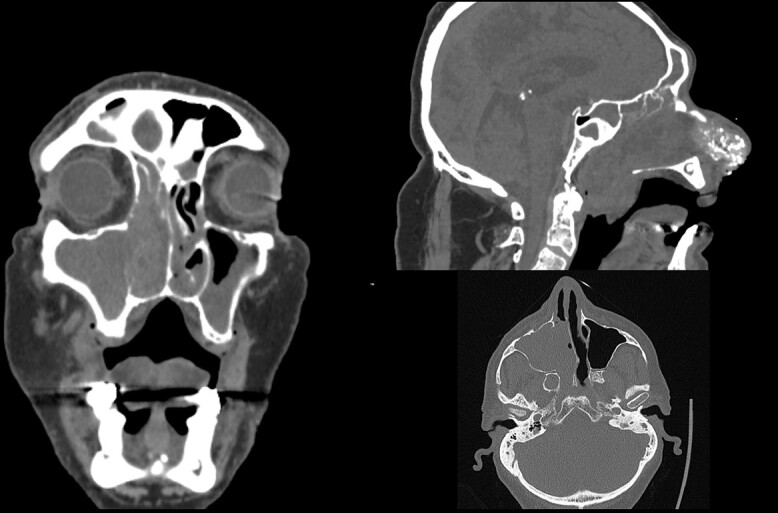
Initial CT scan of the head and neck region in coronal, axial and sagittal view show expansive formation predominantly in the right half of the paranasal sinuses. Right maxillary sinus is completely filled with tumor mass as well as right nasal cavity with infiltration of the septum, all ethmoid cells and the frontal sinus through frontoethmoid recess. Sagittal view show tumor spread in the nasopharynx.

**Figure 2:**
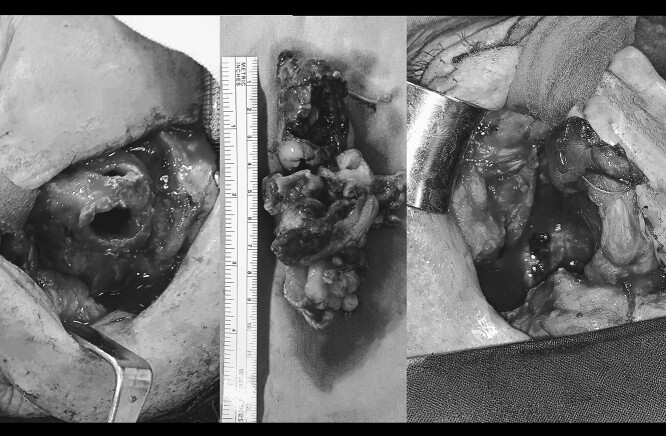
Surgical resection of the tumor by mid-face degloving approach (left).The tumor extended from the lateral wall of the maxillary sinus to the anterior part of the inferior nasal turbinate up to 5 cm below the epipharynx into the oropharynx. Antero-posterior size of the tumor mass was 10 cm. (middle). Surrounding margins were free of tumor cells on the intraoperative frozen section.

A 66-year-old patient with a history of heart disease presented to our hospital with recurrent epistaxis, hyposmia and nasal congestion. On examination, the patient was found with an ulcer-proliferative mass that obstructed the entire right nasal cavity spreading from the epipharynx to the oropharynx. No lymphadenopathy was detectable. Computerized tomography (CT) scan of the head and neck region showed expansive mass primarily in the right maxillary sinus spreading throughout the ostiomeatal complex, infiltrating the septum, all ethmoid cells, entering into the frontal sinus through frontoethmoidal recess and penetrating the nasopharynx. The soft palate was suppressed by the described mass and the initial infiltration of the right pterygoid muscles was also visible (Fig. 1). A biopsy revealed tumor cells built up of a dense mass of small blue cells with round and hyperchromatic nuclei with mitosis. Tumor cells had an ill-defined border and grew into syncytia. Desmoplasia was abundant, with areas of osteoid or chondro-osteoid-like material deposition. Immunohistochemically tumor cells were positive for vimentin, CD99, ERG, FLI-1, MDM2, CDK4, CD56, CK 5/6 and histochemically showed PAS-positive glycogen in the cytoplasm. A working diagnosis leaded to highly malignant and poorly differentiated sarcoma with elements of Ewing’s sarcoma and elements of bone differentiated tumor. Before proceeding with wide surgical resection, the patient received seven cycles of neoadjuvant chemotherapy with paclitaxel and carboplatin. The aggressive tumor growth was stopped, but tumor mass was minimally reduced. Surgical resection of the tumor was performed by mid-face degloving approach with temporary ostectomy of the frontal process of the maxilla and the anterior wall of the maxillary sinus. Medial maxillectomy and resection of the tumor were then performed. The tumor extended from the lateral wall of the maxillary sinus to the anterior part of the inferior nasal turbinate up to 5 cm below the epipharynx into the oropharynx. The posterior part of the septum on the rostrum was removed and bilateral ethmoidectomy was performed. The lamina papyracea was also resected; the skull base was inspected and was completely free of tumor. The part of the nasolacrimal canal that was in contact with the tumor was resected and a small stump was left on the lacrimal sac on which marsupialization was performed. Surrounding margins were free of tumor cells on the intraoperative frozen section (Fig. 2).

**Figure 3:**
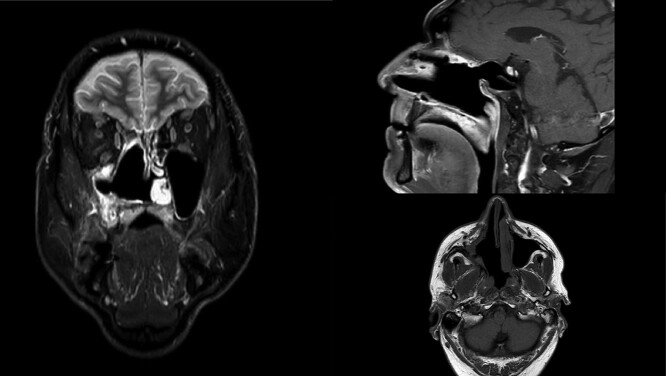
MR scan in coronal, sagittal and axial view on control 14 months postoperatively. Right nasal cavity and right maxillar sinus and ethmoid widely open free of tumor.

The final histopathology corresponded to the biopsy findings. Due to the lack of accurate diagnosis, molecular pathological analysis of all available EWS FLI1 and EWS ERG fusion genes by polymerase chain reaction method was performed and the result was negative. Furthermore, no redistribution of the EWSR1 gene was found by the FISH method, and MDM2 was poorly amplified in 2% of tumor cells, which would exclude the diagnosis of Ewing’s sarcoma. Samples were also sent for consultation and review to the other three Pathology Clinics. Their histopathological findings corresponded to a poorly differentiated high-grade pleomorphic sarcoma with elements indicating bone differentiation and diagnostically corresponded to osteosarcoma, but without definitive diagnosis. The patient was presented again to the board for mesenchymal malignancies and a decision on adjuvant radiotherapy was made. The patient received 60 Gy of radiotherapy in 30 fractions and was free of tumor 14 months postoperatively (Fig. 3). One month later, the patient noticed a small nodule in the right parotid gland. An ultrasound-controlled fine-needle biopsy was performed. The biopsy finding, as well as definitive histopathology diagnose performed after superficial parotidectomy, corresponded to a pleomorphic adenoma.

## DISCUSSION

Head and neck sarcomas are extremely rare tumors that pose a challenge in treatment. Making an accurate diagnosis is often difficult, as seen in our case and in another cases of undifferentiated sphenoid sinus sarcomas, described in the literature that, unfortunately, ended in a fatal outcome [4]. Complete surgical resection with tumor-free margins is the only favorable prognostic factor but due to the proximity of anatomically vital structures and limited space, is not always possible. Tumor management is not facilitated with the rapid and aggressive growth of those tumors and often associated with late clinical presentation. Hence, in different types of head and neck sarcomas, treatment is continued with adjuvant radiotherapy [1, 5–7]. The neoadjuvant therapy, received by our patient, is not part of the regular treatment plan of the cases described in the literature. Most cases, related to different types of head and neck sarcomas, primarily have been treated by surgical resection and adjuvant therapy. A small number of cases were treated only with radiotherapy and chemotherapy [3, 5–9]. In our case, neoadjuvant therapy stopped aggressive tumor growth but did not significantly reduce the tumor mass. Due to the underlying cardiac disease, noncardiotoxic chemotherapy was chosen. The question is whether a different combination of chemotherapeutics would have had a better preoperative outcome or the same was a little bit lacking because the sarcoma was poorly differentiated. The choice of treatment was a major challenge; hence, our patient’s sarcoma had elements of pleomorphic and Ewing’s sarcoma, as well as elements consistent with osteosarcoma.

Although for Ewing’s sarcoma and pleomorphic sarcoma chemoradiation or neoadjuvant chemotherapy is the desirable first-line treatment, for osteosarcoma the results in the literature are still the subject of debate. The Memorial Sloan Kettering Cancer Center’s experience for head and neck osteosarcomas could not conclusively confirm improved tumor local control, decrease presence of distant metastases or improved disease-specific survival with the addition of neoadjuvant chemotherapy to conventional treatment. Furthermore, they suggest that the bone architecture of the tumor does not allow the mass to ‘shrink’ [7, 9, 10].

In patients with the N0 neck, elective neck dissection is not part of the treatment standard because metastases to regional lymph nodes in head and neck sarcoma are relatively rare [1]. The frequency varies according to individual subtypes. Higher incidence of lymph node metastases is associated with clear cell sarcomas, epithelioid sarcomas, synovial sarcomas, angiosarcomas and rhabdomyosarcomas [11].

## CONCLUSION

Making an exact diagnosis of the sarcoma type can be a histopathology dilemma, which makes it difficult to decide on a therapeutic approach. It is clear that surgical excision with clean margins is what we strive for by the oncological postulates, but the anatomical complexity of the region and the extent of the tumor often make such an approach difficult. Therefore, diagnosis at an early stage of tumor disease is imperative. In rare cases, to improve the prognosis of treatment, surgery must be combined with other methods of oncological treatment, chemotherapy and/or radiotherapy as described in our case.

## AUTHORS’ CONTRIBUTIONS

All of the authors have read and approved the manuscript, and all authorship contributions have been verified to adhere to ICMJE guidelines.
